# Paternity Leave, Father Involvement, and Parental Conflict: The Moderating Role of Religious Participation

**DOI:** 10.3390/rel9100289

**Published:** 2018-09-22

**Authors:** Richard J. Petts

**Affiliations:** Department of Sociology, Ball State University, North Quad 222, Muncie, IN 47306, USA; rjpetts@bsu.edu

**Keywords:** paternity leave, fatherhood, religious participation, father involvement, parental conflict

## Abstract

Numerous studies show that taking paternity leave is associated with increased father involvement. However, fewer studies have explored contextual factors that may increase (or diminish) the likelihood that paternity leave-taking provides benefits to families. Using data from the Fragile Families and Child Wellbeing Study, this study examines the associations between paternity leave, fathers’ religious participation, father involvement, and parental conflict, and whether fathers’ religious participation moderates the associations between paternity leave, father involvement, and parental conflict. Results suggest that paternity leave-taking, length of paternity leave, and fathers’ religious participation are associated with increased father involvement but are unrelated to parental conflict. Results also suggest that religious participation may enhance the association between paternity leave and family outcomes; paternity leave-taking and length of paternity leave are only associated with lower levels of parental conflict among families in which fathers attend religious services frequently. Moreover, fathers who take leave and attend religious services frequently are more likely to be involved with their child than fathers who take leave but do not attend religious services.

There has been increased interest in parental leave within the U.S. Six states and Washington, DC have passed legislation providing paid family leave, more companies than ever offer paid parental leave, and most Americans are supportive of paid parental leave (Horowitz et al. 2017; Petts et al. 2018; National Partnership for Women and Families 2018). Relatedly, scholars have also begun to examine the potential consequences of paternity leave-taking for American families, finding that paternity leave is associated with greater father involvement (Petts and Knoester 2018; Pragg and Knoester 2017). Combined with results from European studies that also demonstrate a link between paternity leave and stronger parental relationships (Kotsadam and Finseraas 2011), evidence largely suggests that taking paternity leave and longer periods of leave provides benefits to families.

Despite increased attention to the predictors and potential consequences of paternity leave-taking, more work in this area is needed. For example, scholars have yet to extensively consider the contexts surrounding leave-taking and whether the relationship between paternity leave and family outcomes may vary by these contextual factors. One factor that is important to consider is religion. Religion encourages fathers to be more involved in their family life and provides fathers with social support and guidance that may help them to become more engaged parents and partners (Palkovitz 2002; Wilcox 2004). Indeed, there is evidence suggesting that fathers increase their religious participation after the birth of a child, and religious fathers are more likely to be involved in their children’s lives than nonreligious fathers (King 2003; Petts 2007, 2011). Evidence also suggests that fathers’ religious participation is associated with more favorable relationship outcomes with mothers (Mahoney 2010; Wolfinger and Wilcox 2008). Thus, fathers’ religious participation may moderate the relationships between paternity leave, father involvement, and parental relationships. That is, actively religious fathers may receive greater social support and be more likely to sanctify their family relationships while on leave (Mahoney et al. 2003), which may be associated with more frequent father involvement and fewer parental arguments relative to less religious fathers.

The current study builds on previous research linking paternity leave-taking to father involvement and parental relationships by examining whether fathers’ religious participation moderates these relationships. In doing so, this study contributes to a growing literature on the potential consequences of paternity leave-taking by assessing whether fathers’ religious participation is one contextual factor that may be associated with the degree to which fathers become more invested in their family life after taking leave.

## 1. Conceptual Framework

### 1.1. Paternity Leave

The United States is an outlier in regard to family leave policy; the U.S. is one of only a few countries throughout the world that does not have a statutory paid parental leave entitlement, and most OECD countries also guarantee paid leave to fathers (Blum et al. 2017; Raub et al. 2018). However, the only national leave policy in the U.S. is the Family and Medical Leave Act (FMLA), which allows employees who meet eligibility requirements to take up to 12 weeks of unpaid leave after childbirth (Blum et al. 2017). In addition, four states provide paid family leave to new parents and three additional states have passed legislation to implement paid family leave in the future (National Partnership for Women and Families 2018). Some workers may also have access to employer-based leave programs, but these are relatively rare and are more commonly found in high-paying occupations (Albiston and O’Connor 2016). Overall, the piecemeal structure of leave in the U.S. makes it challenging for many workers to take leave after having a child; 40% of employees are not eligible for leave under FMLA, statutory paid leave is not available to fathers in 46 states, and only 16% of workers have access to paid parental leave from their employer (Blum et al. 2017; Bureau of Labor Statistics 2018).

In addition to lack of access to leave, fathers also face social and cultural barriers to leave-taking. For example, many workplaces are structured around the ideal worker norm that assumes employees prioritize work above all else (Williams 2000). Fatherhood norms also continue to emphasize fathers’ roles as economic providers, despite an increase in men expressing a desire to spend more time with their families (Marsiglio and Roy 2012; McGill 2014). These expectations provide benefits to men within the workplace, but can also lead men who take leave to be stigmatized (Killewald 2013; Rudman and Mescher 2013; Williams et al. 2013). Indeed, taking leave is associated with negative consequences such as lower performance ratings and lower earnings for men (Coltrane et al. 2013; Rege and Solli 2013).

Despite the challenges U.S. fathers face in taking leave, most fathers take at least some time off work after the birth of a child (Petts and Knoester 2018; Pragg and Knoester 2017). Some fathers may use sick or personal days, whereas others may take unpaid time off, but evidence suggests that few fathers use FMLA for paternity leave and less than half of fathers are able to take paid time off (Harrington et al. 2014; Klerman et al. 2012; Petts et al. 2018). Perhaps not surprisingly, American fathers take relatively short periods of leave, with average leaves lasting one week or less (Petts et al. 2018; Petts and Knoester 2018; Pragg and Knoester 2017).

### 1.2. Paternity Leave, Father Involvement, and Parental Conflict

Understanding the current structure and usage of paternity leave in the U.S. is important because it may have implications for fathers’ engagement in their family life. Fathers increasingly state that being actively engaged in their children’s lives is important to them, but they often find it challenging to meet these desires due to expectations at work and pressures of being an economic provider (Aumann et al. 2011; Doucet 2013; McGill 2014). The time off of work provided by paternity leave may enable fathers to act on their desires and be an engaged parent from birth (Rehel 2014; Tanaka and Waldfogel 2007). Fathers may be able to establish early bonds with their child as well as have a focused period of time in which to learn how to be a parent (Rehel 2014; Tanaka and Waldfogel 2007). By having this time, fathers may become more attached to their child as well as become a more confident parent. In doing so, fathers who take paternity leave (and longer leaves) may develop stronger father identities and be perceived as competent parents by themselves and others (Petts and Knoester 2018; Pragg and Knoester 2017; Rehel 2014). As a result, paternity leave may help to facilitate father involvement.

Indeed, evidence from both the U.S. and Europe suggests that fathers who take longer periods of paternity leave are more engaged with their children both in infancy and later in childhood (Haas and Hwang 2008; Huerta et al. 2014; Nepomnyaschy and Waldfogel 2007; Petts and Knoester 2018; Pragg and Knoester 2017; Tanaka and Waldfogel 2007). However, studies have not yet considered contextual factors that may enhance (or diminish) the likelihood that fathers utilize the time provided by paternity leave to become actively involved parents. Since father involvement is associated with positive outcomes for children (Lamb 2010; Sarkadi et al. 2008), it is important to further explore the link between paternity leave and father involvement.

Similarly, relationship conflict has important implications for couples and children. For example, high levels of conflict between partners, and negative communication during conflict, is associated with a higher risk of divorce or separation (Birditt et al. 2010; Carrère and Gottman 1999). Relationship conflict between parents is also associated with lower well-being among children (Grych and Fincham 1990). Thus, it is also important to consider factors that may help to promote parental relationship quality.

Paternity leave-taking may also help to facilitate stronger parental relationships. Similar to the idea that fathers may use the time off provided by paternity leave to focus on their relationship with their new child, fathers may also use this time to focus on their relationship with their coparent. Having a child is a significant life event, and parents may be able to strengthen their relationship with each other by having time together after the birth of a child. Fathers’ use of paternity leave may also enable parents to set parenting expectations and learn to coparent together (Almqvist and Duvander 2014; Bünning 2015; Rehel 2014). Having dedicated time to learn how to divide parenting tasks in a way that both parents will be comfortable with may be particularly important as more couples espouse egalitarian ideals (Gerson 2010; Pedulla and Thébaud 2015), as egalitarianism is positively associated with relationship quality between partners (Carlson et al. 2016; Carlson et al. 2018; Frisco and Williams 2003). There is also evidence that paternity leave-taking increases fathers’ participation in domestic tasks, which is associated with fewer conflicts and an increased likelihood of perceiving the division of household labor as equitable (Almqvist and Duvander 2014; Bünning 2015). Therefore, taking paternity leave (and longer periods of leave) may be associated with stronger parental relationships and lower levels of conflict (Kotsadam and Finseraas 2011).

### 1.3. Religious Participation, Father Involvement, and Parental Conflict

A sizeable body of literature also suggests that religion may promote positive family outcomes (Mahoney 2010; Mahoney et al. 2001). Religious organizations generally emphasize the importance of family life, and provide teachings and guidelines for parents (Edgell 2006; King 2003; Wilcox 2004). Religious institutions may also provide social support for fathers, and encourage them to be engaged parents and partners (Dollahite 1998; Petts 2007). In addition, religion may also provide men with a sense of meaning and purpose in life, as well as a framework for dealing with any stresses associated with becoming a new parent (Ellison and Levin 1998; Palkovitz 2002). Moreover, active involvement in religion may increase the likelihood that fathers sanctify—or place a high level of [spiritual] meaning or significance on—their family relationships (Mahoney et al. 2003). Overall, fathers who attend religious services frequently may be more likely to hear messages about the importance of family life, more likely to feel supported by a religious community, and more likely to internalize these ideas to sanctify family relationships. Consequently, religious participation by fathers may be associated with positive family outcomes such as more frequent father involvement and lower parental conflict.

Indeed, evidence largely suggests that religiously active fathers are more likely to be engaged in their children’s lives than less religious fathers. Specifically, studies suggest that religious fathers report higher quality relationships with their children, are more involved in activities with their child and youth activities more generally, and report higher levels of parental supervision than less religious fathers (Bartkowski and Xu 2000; King 2003; Petts 2007; Roggman et al. 2002; Wilcox 2004). Greater involvement by religious fathers may be due to sanctification, as highly religious fathers feel their role as a parent is “sanctified” and that religion supports their goal of being an involved father (Lynn et al. 2016).

Similarly, religious participation is associated with higher quality, more stable relationships. That is, religious participation is associated with increased marital stability, a lower risk of divorce, and higher relationship satisfaction between partners (Call and Heaton 1997; Mahoney 2010). There is also evidence that fathers’ religious participation, in particular, contributes to higher relationship quality (Wilcox and Wolfinger 2008; Wolfinger and Wilcox 2008). Higher relationship quality and lower levels of conflict among religious parents may also be due, at least in part, to the sanctification of family relationships; viewing relationships as having spiritual significance decreases the likelihood of engaging in hurtful, negative behaviors, and increases the likelihood of working together to resolve conflict (Kusner et al. 2014; Mahoney et al. 1999; Wilcox and Wolfinger 2008).

### 1.4. Religious Participation as a Moderator

In addition to evidence suggesting that religious participation is associated with greater father involvement and lower relationship conflict, it is also possible that religious participation moderates the associations between paternity leave-taking (and length of leave) and father involvement and relationship conflict. That is, among fathers who take leave, father involvement may be higher and parental conflict may be lower for fathers with high levels of religious participation than for fathers with low levels of religious participation. Religiously active fathers may be more likely to sanctify their family relationships (Mahoney et al. 2003). In doing so, actively religious fathers may find greater meaning in these relationships, and may place more importance on interacting with their children and coparents in positive ways than less religious fathers (Mahoney et al. 2003; Mahoney 2005).

Periods of paternity leave are generally short in the U.S. (Petts et al. 2018; Petts and Knoester 2018; Pragg and Knoester 2017), which may leave fathers with only a limited period of time to bond with their child and establish coparenting expectations. By believing that family relationships have spiritual significance, actively religious fathers may be particularly motivated to utilize whatever [little] time they may have while on leave. Because fathers’ religious participation is associated with greater father involvement and higher quality relationships with romantic partners (Bartkowski and Xu 2000; King 2003; Petts 2007; Roggman et al. 2002; Wilcox 2004; Wilcox and Wolfinger 2008; Wolfinger and Wilcox 2008), it is also possible that these associations persist for fathers who take paternity leave.

## 2. Hypotheses

Three hypotheses grounded in the conceptual framework guide this study:

**Hypothesis 1.** Paternity-leave taking and length of paternity leave will be positively associated with father involvement and negatively associated with relationship conflict.
**Hypothesis 2.** Father’s religious participation will be positively associated with father involvement and negatively associated with relationship conflict.
**Hypothesis 3.** The relationships between paternity leave, father involvement, and relationship conflict will be moderated by fathers’ religious participation. That is, among fathers who take paternity leave (and longer periods of leave), fathers with higher levels of religious participation will more involved in their children’s lives, and argue less frequently with mothers, than fathers with lower levels of religious participation.

## 3. Data and Methods

### 3.1. Sample

Data for this study is taken from the Fragile Families and Child Wellbeing Study (FFCW). The FFCW is a longitudinal birth cohort study that follows 4898 children born between 1998 and 2000 and their parents. Fragile families are defined as unmarried parents and their children, and these data consist of an urban sample with high percentages of low-income, minority, and unmarried parents. Data was collected from parents shortly after the birth of their child (W1), and follow-up interviews were conducted when children were approximately one (W2), three (W3), five (W4), nine (W5), and fifteen years old (W6).

For this study, the first two waves are used to assess whether paternity leave is associated with father involvement and parental conflict in the year following a child’s birth, and whether religious participation moderates these relationships. The sample is restricted to families in which mothers and fathers were interviewed in the first two waves, families in which fathers were employed at W1 to be eligible for paternity leave, and families in which fathers answered the questions about leave. These restrictions result in a final sample size of 2109 families.

### 3.2. Paternity Leave

For this study, paternity leave is defined as taking time off for the birth of a child. In the W2 survey, fathers reported on whether they took any time off of work after the birth of the focal child, and how many weeks of leave they took. These questions were used to construct two indicators. Paternity leave-taking indicates whether fathers took leave (1 = *yes*). Length of paternity leave indicates whether fathers took no leave, one week, two weeks, or more than two weeks of leave.

### 3.3. Religious Participation

Fathers reported on how often they attended religious services in the W1 survey. Religious participation indicates whether fathers never attend, hardly attend, attend several times/year, attend several times/month, or attend services at least weekly.

### 3.4. Father Involvement and Parental Relationship Conflict

Father involvement and parental relationship conflict are used as the outcomes of interest in this study because measures are available at both W1 and W2, which helps to account for levels of father involvement and parental conflict before or at the time of the child’s birth. Although other indicators of parental relationships are included in the W2 survey (e.g., relationship quality, coparenting quality), these indicators are not available in the W1 survey.^1^

Father involvement is taken from the W2 survey, and indicates how many days per week fathers reported engaging in eight activities such as reading, singing songs, telling stories, and playing with their child (α = 0.83). The mean is used as the indicator. In the W1 survey, fathers were asked about their involvement in activities prior to the birth of the child. Prenatal involvement indicates whether fathers(a) gave the mother money to buy things for the baby during the pregnancy and (b) helped in other ways such as providing transportation to the prenatal clinic or helping with chores. Parental conflict is taken from the W2 survey, and indicates how often mothers report arguing with fathers about things that are important (1 = *never* to 5 = *always*). In the W1 survey, mothers report how often (1 = *never* to 3 = *often*) they argue with fathers about money, spending time together, sex, the pregnancy, drinking or drug use, and being faithful (α = 0.61). The mean is used as the indicator.

### 3.5. Control Variables

A number of variables taken from W1 were included as controls. Fathers’ religious affiliation is indicated by whether fathers identify as (a) Catholic, (b) conservative Protestant, (c) mainline Protestant,(d) other Protestant, (e) other religious affiliation, or (f) no religious affiliation (used as reference group) using the classification scheme from Steensland et al. (2000) as a guide. Relationship status at W1 is categorized as (a) married (used as reference category), (b) cohabiting, and (c) nonresident father. Controls are also included for fathers’ educational attainment (1 = *did not complete high school* to 4 = *college degree*), fathers’ income (0 = *less than $10,000* to 8 = *$75,000 or more*), mothers’ income (0 = *less than $5000* to 4 = *$20,000 or more*), fathers’ age, whether father was born in the U.S., number of other children, child’s age (at W2, in months), child gender (1 = *male*), and length of time that the mother took off of work following the child’s birth (taken from the W2 survey). Father’s work hours are categorized as (a) part-time (less than 35 h a week), (b) full-time (35–44 h a week, used as reference category), and (c) more than full time (45 h a week or more). Fathers’ occupation type is categorized as(a) professional, (b) labor (used as reference category), (c) service, (d) sales, or (e) other occupational type. Father’s race/ethnicity is coded as (a) White (used as reference category), (b) Black, (c) Latino, or (d) other race/ethnicity. Three additional controls assess fathers’ attitudes. Positive father attitudes are indicated by fathers’ level of agreement at (1 = *strongly disagree* to 4 = *strongly agree*) on whether (a) being a father and raising children is one of the most fulfilling experiences for a man, (b) I want people to know that I have a new child, and (c) not being a part of my child’s life would be one of the worst things that could happen to me (α = 0.73). The mean response is used. Fathers were also asked to identify which fathering role (provide regular financial support, teach child about life, provide direct care, show love and affection to the child, provide protection for the child, or serve as an authority figure and discipline the child) was most important, and engaged father attitudes indicates fathers who identified either providing direct care or showing love and affection to the child as most important. Traditional gender attitudes indicates whether fathers agree that it is much better for everyone if the man earns the main living and the woman takes care of the home and family.

### 3.6. Analytic Strategy

Ordinary least squares (OLS) regression models were used in this study. First, OLS models were used to assess whether paternity leave-taking, length of paternity leave, and father’s religious participation are associated with father involvement and parental conflict in separate models. Second, interaction terms were included in separate models to assess whether the relationships between paternity leave, father involvement, and parental conflict were moderated by religious participation. Missing data were accounted for using multiple imputation, and combined results from ten imputed models are presented here.

## 4. Results

Summary statistics for all variables are reported in Table 1. Consistent with previous research, most fathers take paternity leave (79%), but take short leaves, on average (approximately one week). Mean values also suggest that fathers engage in activities with their child approximately four days a week (M = 4.35), mothers report arguing with fathers between “rarely” and “sometimes” (M = 2.80), and fathers report attending religious services several times a year on average (M = 1.91).

Results examining the relationships between paternity leave, religious participation, and father involvement are presented in Table 2. Consistent with the first two hypotheses, paternity leave and religious participation are both positively associated with father involvement. As shown in Model 1, taking paternity leave is associated with engaging in activities with children just under one-half day more frequently per week compared to not taking leave (*b* = 0.40, *p* < 0.001). Similarly, as shown in Model 3, longer periods of paternity leave are associated with more frequent father involvement (*b* = 0.23, *p* < 0.001). Moreover, as shown in both Models 1 and 3, more frequent attendance at religious services is associated with more frequent involvement with young children (*b* = 0.06, *p* < 0.05).

**Figure 1.**
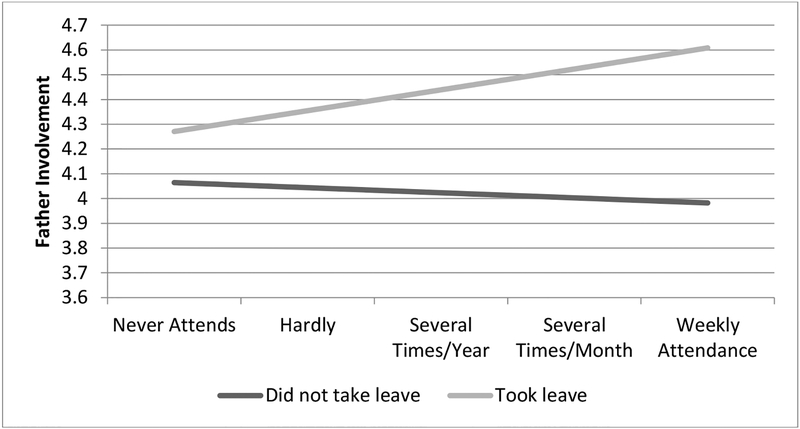
Predicted values of the relationships between paternity leave-taking, religious participation, and father involvement

Results assessing whether religious participation moderates the relationships between paternity leave and father involvement are included in Models 2 and 4 of Table 2. Although the interaction term for length of paternity leave and religious participation is not significant (Model 4), there is some evidence that religious participation moderates the relationship between paternity leave-taking and father involvement (Model 2). Consistent with hypothesis 3, religious participation is especially likely to be associated with greater father involvement among fathers who take paternity leave (*b* = 0.11, *p* < 0.10), although this interaction term is only marginally significant. This relationship is illustrated in Figure 1, which shows predicted values of father involvement calculated from the estimates in Model 2 of Table 2. As shown in Figure 1, more frequent attendance at religious services is associated with more frequent involvement among fathers who take paternity leave. In contrast, among fathers who do not take paternity leave, religious participation is largely unrelated to father involvement.

**Figure 2.**
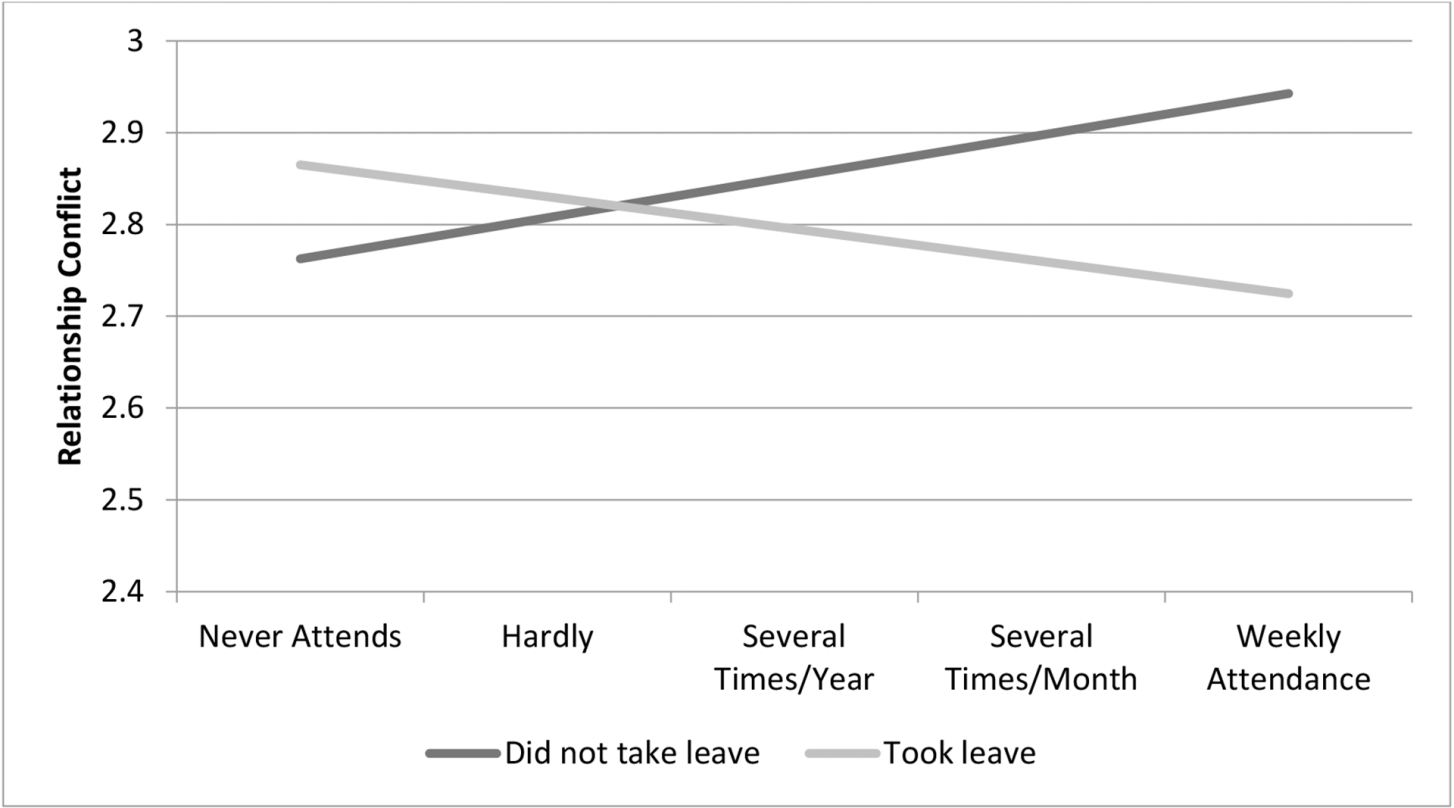
Predicted values of the relationships between paternity leave-taking, religious participation, and relationship conflict.

Results examining the relationships between paternity leave, religious participation, and parental conflict are presented in Table 3. In contrast to the first two hypotheses, none of the key variables—paternity leave-taking, length of paternity leave, and religious participation—are associated with parental conflict, as shown in Models 1 and 3. However, when interaction terms are included in Models 2 and 4, there is some evidence in support of hypothesis 3. First, as shown in Model 2, religious participation is especially likely to be associated with less frequent conflict within families in which fathers take paternity leave (*b* = −0.08, *p* < 0.05). This relationship is illustrated in Figure 2. As shown in Figure 2, more frequent attendance at religious services is associated with less frequent conflict when fathers take paternity leave. In contrast, when fathers do not take paternity leave, religious participation is positively associated with parental conflict.

**Figure 3.**
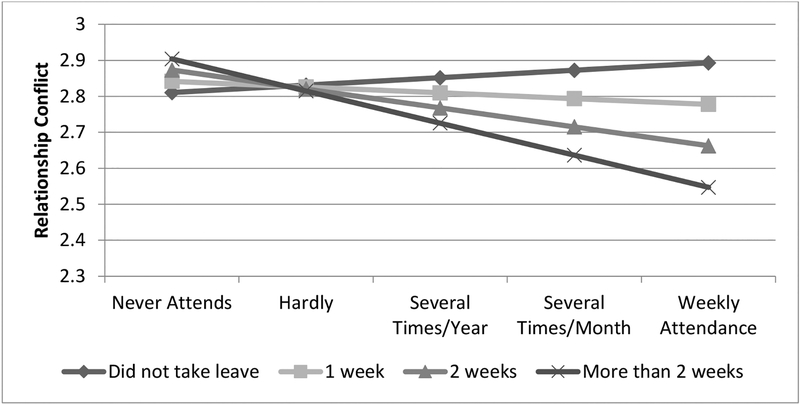
Predicted values of the relationships between length of paternity leave, religious participation, relationship conflict.

In addition, as shown in Model 4 of Table 3, there is evidence that religious participation moderates the relationship between length of paternity leave and parental conflict (b = −0.04, p < 0.10), although this relationship is only marginally significant. This relationship is illustrated in Figure 3. As shown in Figure 3, religious participation is associated with a slight increase in parental conflict when fathers do not take leave. In contrast, more frequent religious participation is associated with less frequent conflict when fathers take paternity leave, with longer periods of leave being associated with lower levels of conflict when religious participation is higher.

## 5. Discussion

Despite increased attention on paternity leave in the U.S., few studies have focused on factors that may contextualize the associations between paternity leave-taking and family outcomes. This study advances our understanding of factors that may contextualize the relationships between paternity leave, father involvement, and parental conflict by focusing on fathers’ religious participation. Overall, results provide some evidence suggesting that paternity leave-taking (and length of leave) and fathers’ religious participation are independently associated with father involvement, and also that fathers’ religious participation moderates the relationships between paternity leave, father involvement, and parental conflict.

Consistent with previous research, results from this study suggest that paternity leave-taking and length of paternity leave are associated with more frequent father involvement (Haas and Hwang 2008; Huerta et al. 2014; Nepomnyaschy andWaldfogel 2007; Petts and Knoester 2018). Having time off when a child is born may provide fathers with time to feel comfortable performing parenting tasks as well as to bond with their newborn child (Rehel 2014; Tanaka andWaldfogel 2007). These early experiences may increase attachments between fathers and children, strengthening men’s identities as fathers and increasing the likelihood that fathers maintain a high level of involvement throughout the child’s life (Cabrera et al. 2008; Petts and Knoester 2018).

However, results from this study did not support the hypothesis that paternity leave-taking (and length of leave) would be associated with parental conflict. The transition to parenthood (or having an additional child) is often challenging, as parents need to adapt to their new roles (Twenge et al. 2003). Consequently, parental relationship quality declines after having a new child and conflict often increases (Cowan et al. 1985; Twenge et al. 2003). Although paternity leave may provide parents with time to figure out how to coparent together, it may also provide additional time for parents to argue with each other as they figure out their new roles. As such, any positive benefits of paternity leave for parents (such as symbolizing a commitment by fathers to be an engaged parent) may be offset by additional time to experience conflict. This may also help to explain why relatively few studies have found an influence of paternity leave on parental relationships (Kotsadam and Finseraas 2011), whereas a large body of literature has noted the benefits of paternity leave (and length of leave) for father involvement.

Similar to the findings for paternity leave (and length of leave), results from this study also showed that fathers’ religious participation is positively associated with father involvement but unrelated to parental conflict. Consistent with previous research, actively religious fathers may receive messages about the importance of family life as well as social support that encourages and enables them to be involved parents (King 2003; Petts 2007; Wilcox 2004). Thus, fathers who attend religious services frequently may be motivated to be engaged in their children’s lives early on, as evidence from this study and previous research suggests (Bartkowski and Xu 2000; Petts 2007; Roggman et al. 2002). However, although previous research suggests that religious participation is associated with lower family conflict (Mahoney et al. 1999; Wilcox and Wolfinger 2008), this relationship may be offset by the increases in conflict that often occur after having a new child (Cowan et al. 1985; Twenge et al. 2003).

It is also somewhat surprising that religious affiliation was unrelated to both father involvement and parental conflict. There was some evidence that Catholic fathers were more involved in their children’s lives than unaffiliated fathers and that conservative Protestant fathers reported less parental conflict than religiously unaffiliated fathers (*p* < 0.10) in supplementary analyses, but these variations disappeared after accounting for fathers’ religious participation. As such, religious participation appears to be more important than affiliation in these data. Yet, it is also possible that the lack of significant findings on religious affiliation may be due to competing expectations for religiously affiliated fathers (especially fathers who are more religiously conservative). That is, religiously conservative fathers may be perceived as the spiritual head of the household and thus may place particular importance on sanctifying family relationships (Gallagher and Smith 1999). However, conservative denominations are also more likely to encourage traditional gender roles within families, which may reduce the likelihood that fathers are highly engaged parents (DeMaris et al. 2011; Denton 2004). These competing expectations may offset one another, resulting in no relationship between religious affiliation, father involvement, and parenting conflict. Future research should further explore variations by religious affiliation.

Although there were no direct associations between paternity leave or fathers’ religious participation and parental conflict, there is some evidence that the association between paternity leave and parental conflict varies by how frequently fathers attend religious services. At low levels of religious participation, paternity leave-taking and length of paternity leave are largely unrelated to parental conflict. In contrast, when fathers attend religious services frequently, taking paternity leave (and longer periods of leave) is associated with lower levels of parental conflict. Specifically, among fathers who attend religious services weekly, there is approximately a 1/3 standard deviation difference in parental conflict between those who take more than two weeks of leave and those who do not take leave.

Similarly, there is also some evidence that fathers’ religious participation may enhance the association between paternity leave-taking and father involvement (although this relationship is only marginally significant). Although fathers who take paternity leave report higher levels of father involvement regardless of how frequently they attend religious services, the gap in involvement between fathers who take leave and those who do not increases at higher levels of religious participation. Specifically, among fathers who attend religious services weekly, those who take leave engage with their child over one-half day more frequently (0.4 standard deviations) than fathers who do not take leave.

These findings suggest that religious participation may be one contextual factor that may motivate fathers to become more invested in their family life while on leave. Fathers who attend religious services frequently may sanctify their family relationships and find more meaning in learning and performing parenting tasks after the birth of a new child compared to less religious fathers (Mahoney et al. 2003). Actively religious fathers may also be more willing to collaborate with mothers during this time, and perhaps focus more on the positive aspects of being a new parent as opposed to the frustrations and stresses that can also occur (Mahoney 2005; Mahoney et al. 1999). As such, fathers who attend religious services frequently may have fewer arguments with mothers while on leave compared to fathers who attend religious services less frequently. Due to the lack of a national paid family leave policy as well as other barriers to leave-taking for fathers, most fathers take relatively short periods of time off when a child is born (Albiston and O’Connor 2016; Petts et al. 2018). Results from this study suggest that involvement in a religious community may encourage fathers to make the most of the limited time at home they have, resulting in an increased likelihood of being engaged in their child’s life one year later and a decreased likelihood of arguing with mothers (Bartkowski and Xu 2000; King 2003; Wilcox and Wolfinger 2008).

There are also some limitations to acknowledge in this study. First, the data contains limited information about how fathers were able to take time off for the birth of their child. Fathers in these data may be using workplace paternity (or parental) leave programs, unpaid time off through FMLA, or other forms of leave (using sick, personal, or vacation days). The publicly available data also does not contain information about the state or region that respondents reside in, which is important given state variations in paid family leave. Knowing exactly what type of leave fathers have access to and are using is essential to get an accurate assessment of the consequences of paternity leave, as well as to better understand potential barriers to paternity leave-taking.

Second, the moderating influence of religious participation on the associations between paternity leave, father involvement, and parental conflict is speculated to be due to religiously active fathers sanctifying their family relationships. Unfortunately, the indicators of religiosity available in the data are limited, and there are no questions that allow for an assessment of the role sanctification plays in these processes. or the extent to which fathers are exposed to religious messages about the importance of family life. Future studies should incorporate additional measures of religiosity—and measures of sanctification in particular—to better understand whether and how religion shapes fathers’ decisions and actions while on paternity leave.

Third, this study focuses on a sample of urban families that are relatively disadvantaged. Although this dataset has been used extensively to study various outcomes associated with fathers’ religious participation (e.g., Petts 2007, 2011; Wolfinger and Wilcox 2008), the findings uncovered here may differ for fathers within different socioeconomic and regional contexts. Future studies should explore the role of religion in decisions about, and consequences of, paternity leave in other contexts (rural, higher SES, etc.).

Despite these limitations, this study contributes to the areas of religion and family life by exploring whether fathers’ religious participation moderates the associations between paternity leave, father involvement, and parental conflict. Although previous studies have found evidence that paternity leave-taking and religious participation may both be associated with family outcomes, this study is the first to examine whether fathers’ religious participation may contextualize the experience of paternity leave. Overall, there is some evidence suggesting that taking paternity leave, and longer periods of leave, is more likely to be associated with more frequent father involvement and lower parental conflict among fathers who attend religious services frequently than among fathers who attend less frequently. Future studies should continue to examine the intersection of family and religion in predicting family outcomes, and the role religion may play in fathers’ decisions about, and behaviors during, paternity leave to better understand the context of paternity leave within the United States.

## Figures and Tables

**Table 1.** Summary Statistics at W1.

Variables	*M*	*SD*	Min	Max
*Dependent Variables*				
Father Involvement	4.35	1.59	0	7
Relationship Conflict	2.80	0.90	1	5
*Key Variables*				
Paternity Leave	0.79	-	0	1
Length of Paternity Leave	1.07	0.78	0	3
Religious Participation	1.91	1.35	0	4
*Controls*				
Catholic	0.30	-	0	1
Conservative Protestant	0.27	-	0	1
Mainline Protestant	0.05	-	0	1
Other Protestant	0.18	-	0	1
Other Religious Affiliation	0.09	-	0	1
No Religious Affiliation *	0.11	-	0	1
Married *	0.34	-	0	1
Cohabiting	0.41	-	0	1
Nonresident	0.25	-	0	1
Number of Other Children	1.02	1.18	0	5
Education	2.31	1.00	1	4
Works Part-Time	0.08	-	0	1
Works Full-Time *	0.49	-	0	1
Works more than Full-Time	0.43	-	0	1
Professional Occupation	0.17	-	0	1
Labor Occupation *	0.49	-	0	1
Sales Occupation	0.08	-	0	1
Service Occupation	0.24	-	0	1
Other Occupation	0.02	-	0	1
Income	3.36	2.18	0	8
Mother’s Income	1.17	1.63	0	4
Age	28.18	6.93	18	57
White *	0.27	-	0	1
Black	0.42	-	0	1
Latino	0.26	-	0	1
Other Race	0.05	-	0	1
U.S. Native	0.84	-	0	1
Child Age	15.60	3.90	5	30
Child is Male	0.52	-	0	1
Length of Maternity Leave	2.46	3.14	0	12
Positive Father Attitudes	3.76	0.40	1	4
Engaged Father Attitudes	0.66	-	0	1
Traditional Gender Attitudes	0.39	-	0	1
Prenatal Involvement	0.93	-	0	1
W1 Relationship Conflict	1.40	0.35	1	3

*N* = 2109.

* Used as reference category.

**Table 2.** Results from OLS regression models predicting father involvement.

Variable	1		2		3		4	
	*b*	SE *b*	*B*	SE *b*	*b*	SE *b*	*b*	SE *b*
Paternity Leave-Taking	0.40	0.09 ***	0.21	0.14				
Length of Paternity Leave					0.23	0.04 ***	0.16	0.08 *
Religious Participation	0.06	0.03 *	−0.02	0.06	0.06	0.03 *	0.02	0.04
Catholic	0.18	0.13	0.18	0.13	0.19	0.13	0.19	0.13
Conservative Protestant	0.00	0.13	0.01	0.13	0.01	0.13	0.02	0.13
Mainline Protestant	−0.04	0.18	−0.04	0.18	−0.04	0.18	−0.04	0.18
Other Protestant	0.00	0.13	0.01	0.13	0.02	0.13	0.03	0.13
Other Religious Affiliation	0.12	0.16	0.12	0.16	0.12	0.16	0.13	0.16
Cohabiting	−0.10	0.09	−0.09	0.09	−0.09	0.09	−0.08	0.09
Nonresident	−0.72	0.11 ***	−0.71	0.11 ***	−0.71	0.11 ***	−0.71	0.11 ***
Number of Other Children	−0.09	0.03 **	−0.09	0.03 **	−0.09	0.03 **	−0.09	0.03 **
Education	−0.01	0.04	−0.01	0.04	−0.02	0.04	−0.02	0.04
Works Part-Time	−0.21	0.13	−0.21	0.13	−0.22	0.13	−0.21	0.13
Works more than Full-Time	−0.01	0.08	−0.01	0.08	−0.01	0.08	−0.00	0.08
Professional Occupation	0.05	0.11	0.06	0.11	0.03	0.11	0.03	0.11
Sales Occupation	0.17	0.13	0.17	0.13	0.18	0.13	0.18	0.13
Service Occupation	−0.03	0.08	−0.03	0.08	−0.05	0.08	−0.05	0.08
Other Occupation	−0.22	0.26	−0.23	0.26	−0.24	0.26	−0.25	0.26
Income	−0.01	0.02	−0.01	0.02	−0.01	0.02	−0.01	0.02
Mother’s Income	0.01	0.02	0.01	0.02	0.01	0.02	0.01	0.02
Age	−0.00	0.01	−0.00	0.01	−0.00	0.01	−0.00	0.01
Black	0.08	0.10	0.07	0.10	0.07	0.10	0.07	0.10
Latino	0.01	0.11	0.01	0.11	−0.01	0.11	−0.01	0.11
Other Race	0.10	0.18	0.10	0.18	0.07	0.18	0.07	0.18
U.S. Native	0.35	0.11 **	0.35	0.11 **	0.35	0.11 **	0.35	0.11 **
Child Age	−0.00	0.01	−0.00	0.01	−0.00	0.01	−0.00	0.01
Child is Male	0.07	0.07	0.07	0.07	0.07	0.07	0.07	0.07
Length of Maternity Leave	0.01	0.01	0.01	0.01	0.01	0.01	0.01	0.01
Positive Father Attitudes	0.26	0.08 **	0.26	0.08 **	0.25	0.08 **	0.25	0.08 **
Engaged Father Attitudes	0.08	0.07	0.08	0.07	0.08	0.07	0.08	0.07
Traditional Gender Attitudes	0.00	0.07	0.00	0.07	0.02	0.07	0.02	0.07
Prenatal Involvement	0.59	0.14 ***	0.59	0.14 ***	0.61	0.14 ***	0.61	0.14 ***
Leave-Taking × Religious Participation			0.11	0.06 †				
Length of Leave × Religious Participation							0.04	0.03
*R*^2^	0.10		0.10		0.11		0.11	

*N* = 2109.

† *p* < 0.10.

* *p* < 0.05.

** *p* < 0.01.

*** *p* < 0.001.

**Table 3.** Results from OLS regression models predicting relationship conflict.

Variable	1		2		3		4	
	*b*	SE *b*	*b*	SE *b*	*b*	SE *b*	*b*	SE *b*
Paternity Leave-Taking	−0.04	0.05	0.10	0.08				
Length of Paternity Leave					−0.04	0.03	0.03	0.05
Religious Participation	−0.02	0.02	0.04	0.03	−0.02	0.02	0.02	0.^.^03
Catholic	−0.02	0.08	−0.02	0.08	−0.02	0.08	−0.02	0.08
Conservative Piotestant	−0.09	0.08	−0.10	0.08	−0.09	0.08	−0.09	0.08
Mainline Protestant	−0.09	0.11	−0.10	0.11	−0.09	0.11	−0.10	0.11
Other Protestant	−0.02	0.09	−0.03	0.09	−0.03	0.09	−0.03	0.09
Other Religious Affiliatien	−0.12	0.09	−0.13	0.09	−0.12	0.09	−0.13	0.09
Cohabiting	0.09	0.06	0.09	0.06	0.09	0.06	0.08	0.06
Nonresident	0.15	0.06*	0.15	0.06*	0.15	0.06*	0.15	0.06*
Number of Other Children	0.01	0.02	0.01	0.02	0.01	0.02	0.01	0.02
Edutation	0.01	0.03	0.01	0.03	0.01	0.03	0.01	0.03
Works Part-Time	0.05	0.08	0.05	0.08	0.05	0.08	0.05	0.08
Works more than Full-Time	−0.01	0.06	−0.01	0.06	−0.01	0.06	−0.02	0.06
Professional Occupation	−0.04	0.07	−0.04	0.07	−0.03	0.07	−0.03	0.07
Sales Occupation	−0.13	0.07 †	−0.13	0.07 †	−0.13	0.07 †	−0.14	0.07 †
Service Occupation	−0.05	0.05	−0.05	0.05	−0.05	0.05	−0.05	0.05
Other Occupation	0.16	0.15	0.16	0.15	0.16	0.15	0.16	0.15
Income	−0.00	0.01	−0.00	0.01	−0.00	0.01	−0.00	0.01
Mother’s Income	0.01	0.01	0.01	0.01	0.01	0.01	0.01	0.01
Age	−0.01	0.00	−0.01	0.00	−0.01	0.00	−0.01	0.00
Black	0.06	0.06	0.07	0.06	0.06	0.06	0.07	0.06
Latino	0.01	0.07	0.01	0.07	0.01	0.07	0.01	0.07
Other Race	0.05	0.15	0.04	0.15	0.05	0.15	0.05	0.15
U.S. Native	0.01	0.06	0.01	0.06	0.01	0.06	0.00	0.06
Child Age	0.00	0.01	0.00	0.01	0.00	0.01	0.00	0.01
Child is Male	−0.04	0.04	−0.04	0.04	−0.04	0.04	−0.04	0.04
Length of Maternity Leave	−0.01	0.01	−0.01	0.01	−0.01	0.01	−0.01	0.01
Positive Father Attitudes	0.01	0.05	0.01	0.05	0.01	0.05	0.01	0.05
Engaged Father Attitudes	−0.04	0.04	−0.04	0.04	−0.04	0.04	−0.04	0.04
Traditional Gender Attitudes	0.08	0.04 †	0.08	0.04 *	0.08	0.04 †	0.08	0.04 †
Prenatal Involvement	−0.02	0.09	−0.02	0.09	−0.02	0.09	−0.02	0.09
W1 Relationship Conflict	0.63	0.06 ***	0.62	0.06 ***	0.62	0.06***	0.62	0.06***
Leave-Taking × Religious Participation			−0.08	0.04*				
Length of Leeve × Riligiouo Participation							−0.04	0.02 †
*R*^2^	0.10		0.10		0.10		0.10	

N = 2109.

† *p* < 0.10.

* *p* < 0.05.

** *p* < 0.01.

*** *p* < 0.001.

## Footnotes

1 These variables were included in supplementary analyses. Although there were instances in which paternity leave and/or religious participation were associated with these outcomes, the relationship between paternity leave and these outcomes did not vary by religious participation.
